# Occurrence of ferredoxin:NAD^+^ oxidoreductase activity and its ion specificity in several Gram-positive and Gram-negative bacteria

**DOI:** 10.7717/peerj.1515

**Published:** 2016-01-11

**Authors:** Verena Hess, Rene Gallegos, J Andrew Jones, Blanca Barquera, Michael H Malamy, Volker Müller

**Affiliations:** 1 Molecular Microbiology and Bioenergetics, Goethe University Frankfurt, Frankfurt am Main, Germany; 2 Department of Molecular Biology and Microbiology, Tufts University, School of Medicine and Sackler School of Graduate Biomedical Sciences, Boston, Masachusetts, United States; 3 Department of Chemical and Biological Engineering, Rensselaer Polytechnic Institute, Troy, New York, United States; 4 Department of Biological Sciences and Center for Biotechnology and Interdisciplinary Studies, Rensselaer Polytechnic Institute, Troy, New York, United States

**Keywords:** Rnf, Energy conservation, Ion pump, Fdred:NAD^+^ oxidoreductase

## Abstract

A ferredoxin:NAD^+^ oxidoreductase was recently discovered as a redox-driven ion pump in the anaerobic, acetogenic bacterium *Acetobacterium woodii*. The enzyme is assumed to be encoded by the *rnf* genes. Since these genes are present in the genomes of many bacteria, we tested for ferredoxin:NAD^+^ oxidoreductase activity in cytoplasmic membranes from several different Gram-positive and Gram-negative bacteria that have annotated *rnf* genes. We found this activity in *Clostridium tetanomorphum*, *Clostridium ljungdahlii*, *Bacteroides fragilis, and Vibrio cholerae* but not in *Escherichia coli* and *Rhodobacter capsulatus*. As in *A. woodii*, the activity was Na^+^-dependent in *C. tetanomorphum* and *B. fragilis* but Na^+^-independent in *C. ljungdahlii* and *V. cholerae*. We deleted the *rnf* genes from *B. fragilis* and demonstrated that the mutant has greatly reduced ferredoxin:NAD^+^ oxidoreductase activity. This is the first genetic proof that the *rnf* genes indeed encode the reduced ferredoxin:NAD^+^ oxidoreductase activity.

## Introduction

Like any other cell, microorganisms use two energy currencies to couple exergonic catabolic reactions to endergonic anabolic reactions, ATP and a transmembrane electrochemical ion gradient. Both are connected by the ATP synthase, a key element in cellular bioenergetics present in all domains of life. Under “respiratory” conditions, the ATP synthase is driven by the electrochemical ion gradient across the membrane to synthesize ATP (Boyer, Cross & Momsen, 1973). The coupling ion is a proton in most cases but some bacteria and archaea use Na^+^ instead of H^+^ as the coupling ion (Grüber et al., 2014; Müller et al., 2005). The reaction is freely reversible and under fermentative conditions ATP hydrolysis drives the generation of an electrochemical ion gradient across the membrane. Bacteria and archaea have evolved a fascinating repertoire of enzymes that generate the electrochemical ion gradient. The respiratory chain of many aerobic bacteria is the same as in mitochondria, i.e. involves complexes I, II, III, and IV (Schägger, 2002). The redox span covered by these complexes is from −320 mV (NADH/NAD^+^) to +800 mV (O_2_/H_2_O) and the energy released is sufficient to synthesize at least three molecules of ATP. In contrast, the facultative anaerobe *Escherichia coli* has a different composition and lacks a complex III (Anraku & Gennis, 1987).

Many strictly anaerobic bacteria such as, for example fermenting clostridia, acetogens or non-acetogens do not even have one of these complexes but rely on other enzyme systems to energize the membrane. In recent years, a novel type of ion-translocating redox reaction was discovered. Inverted membrane vesicles of the anaerobic acetogenic bacterium *Acetobacterium woodii* catalyzed electron transfer from reduced ferredoxin to NAD^+^ with a primary and electrogenic export of Na^+^ from the cytoplasm to the exterior of the cell (Biegel & Müller, 2010). Early experiments designed to verify a sodium ion dependence failed, likely due to the fact that an artificial electron donor was used in the enzymatic assay. Later, the CO dehydrogenase (CODH) was purified from *A. woodii.* This enzyme is able to reduce the ferredoxin purified from *Clostridium pasteurianum* (Hess, Schuchmann & Müller, 2013). Using *C. pasteurianum* ferredoxin reduced by CODH from *A. woodii* as electron donor, a clear dependence of ferredoxin-dependent NAD^+^ reduction on Na^+^ was observed (Hess, Schuchmann & Müller, 2013). Using the same assay, the presence of a sodium ion-dependent ferredoxin:NAD^+^ oxidoreductase activity was demonstrated in another acetogen, *Eubacterium limosum* (Jeong et al., 2015).

Moreover, using inverted membrane vesicles of *A. woodii*, it was demonstrated that the reaction is reversible: ATP hydrolysis drives the generation of a transmembrane electrochemical sodium ion gradient that then drives endergonic, reverse electron flow from NADH to ferredoxin (Hess, Schuchmann & Müller, 2013). The forward reaction is essential for growth of *A. woodii* on H_2_ + CO_2_ and fructose, but the reverse reaction is also essential, although under different growth conditions. During heterotrophic growth on many substrates such as 2,3-butanediol, lactate or ethanol, NADH is the only reductant generated. However, reduced ferredoxin is essential for the operation of the Wood-Ljungdahl pathway (Ragsdale, 2008; Schuchmann & Müller, 2014) and under these conditions, ferredoxin is reduced with NADH as reductant at the expense of the transmembrane electrochemical Na^+^ gradient generated by hydrolysis of ATP synthesized by substrate level phosphorylation (Hess et al., 2015; Weghoff, Bertsch & Müller, 2015). *A. woodii* is a prime example of an organism in which the ferredoxin:NAD^+^ oxidoreductase activity is essential in both directions, depending on the substrate used for growth.

The enzyme mediating the ferredoxin:NAD^+^ oxidoreductase activity was partially purified and amino acid sequences were derived, which allowed to identify the encoding genes. Interestingly, the encoding genes were annotated as *rnf* genes, based on their similiarity to the *rnf* genes of *Rhodobacter capsulatus* (Biegel, Schmidt & Müller, 2009). Deletion of the *rnf* genes in this phototrophic, diazotrophic bacterium led to the inability of the bacterium to fix nitrogen (Schmehl et al., 1993). The similarity of the Rnf proteins to subunits of the membrane-integral, ion translocating NADH:quinone-oxidoreductase (Biegel et al., 2011; Kumagai et al., 1997) led the authors to speculate that the Rnf proteins may constitute a membrane-integral protein complex involved in nitrogen fixation (Schmehl et al., 1993). The nitrogenase reaction requires low potential electrons that can only be generated from NADH at the expense of energy and it was speculated that the Rnf complex catalyzes this endergonic electron transfer with the energy derived from the transmembrane electrochemical ion gradient. Thus, in *R. capsulatus*, energy conservation is by photosynthetic electron transport phosphorylation and the Rnf complex is thought to be involved in anabolic reactions by providing low potential electrons for biosynthetic reactions (Schmehl et al., 1993).

*rnf* genes are widely distributed in bacteria and are also present in a few methanogenic archaea (Biegel et al., 2011). Rnf complexes thus far have not been purified from any source and thus, the final biochemical proof that they are indeed ion-translocating membrane proteins is still missing. Moreover, the anticipated reaction catalyzed by the complex, electron transfer from reduced ferredoxin to the acceptor NAD^+^ has only been reported for *A. woodii* and *C. tetanomorphum* (Biegel et al., 2011; Boiangiu et al., 2005). Despite the obvious lack of experimental data with a purified enzyme, as well as the experimental proof that Rnf in the methanogen *Methanosarcina acetivorans* uses not NAD^+^ but an alternative electron acceptor (Schlegel et al., 2012), the presence of *rnf* genes in bacteria is often taken as indication that these organisms have an ion-translocating ferredoxin:NAD^+^ oxidoreductase. To shed more light on the function of the Rnf complexes in different bacteria, we have isolated the cytoplasmic membranes of several organisms and assayed them for ferredoxin:NAD^+^ oxidoreductase activity. In addition, we determined the ion specificity of the reaction in the different organisms and will present genetic evidence that the ferredoxin:NAD^+^ oxidoreductase activity in *B. fragilis* is indeed catalyzed by the Rnf complex.

## Materials and Methods

### Bacterial strains and growth conditions

*A. woodii* DSM 1030 was grown anaerobically at 30 °C on either 20 mM fructose, 20 mM 2,3-butanediol, 50 mM ethanol, or 80 mM D,L-lactate as carbon source in 4 × 500 ml medium as described previously (Heise, Müller & Gottschalk, 1992). *Clostridium ljungdahlii* DSM 13528 was grown at 37 °C on 56 mM fructose as carbon source in either 2 × 500 ml (for the preparation of membranes) or 20 l (for the preparation of inverted membrane vesicles) complex medium that was prepared as described (Tanner, Miller & Yang, 1993) with slight modifications: 1000 ml medium contained: 20 g MES, 0.5 g Cysteine-HCl, 0.5 g yeast extract, 10 g fructose, 0.25 g KH_2_PO_4_, 2.5 g NH_4_Cl, 0.1 g CaCl_2_ × 2 H_2_O, 0.25 g KCl, 0.5 g MgSO_4_ × 7 H_2_O, 2 g NaCl, 20 mg nitrilotriacetic acid, 10 mg MnSO_4_ × H_2_O, 8 mg Fe(NH_4_)(SO_4_)_2_ × 6 H_2_O, 2 mg CoCl_2_, 10 mg ZnSO_4_ × 7 H_2_O, 0.2 mg CuCl_2_, 2 mg NiCl_2_ × 6 H_2_O, 0.2 mg Na_2_MoO_4_ × 2 H_2_O, 1 mg Na_2_SeO_4_ × 5 H_2_O, 2 mg Na_2_WO_4_, 20 μg biotin, 50 μg Ca-panthothenat, 20 μg folic acid, 50 μg liponic acid, 50 μg nicotinic acid, 100 μg pyridoxine-HCl, 50 μg *p*-aminobenzoic acid, 50 μg riboflavin, 50 μg thiamin-HCl, 1 μg vitamin B_12_, 1 mg resazurin, pH 6.5. *Clostridium tetanomorphum* DSM 4474 was grown at 37 °C on 222 mM glutamate in 2 × 500 ml medium that was prepared as described (Jayamani, 2008). *E. coli* BL21(DE3) was grown at 37 °C in 1,500 ml LB medium (Sambrook, Fritsch & Maniatis, 1989) under rigorous shaking (150 rpm). *R. capsulatus* SB1003 was grown at 30 °C under diazotrophic conditions in 4 × 500 ml medium, either phototrophic in N-free RCVB medium (Weaver, Wall & Gest, 1975) containing 30 mM malate as carbon source or respiratory in the dark in N-free CA medium (Saeki & Kumagai, 1998) containing 20 mM glucose and 80 mM DMSO. *Bacteroides fragilis* was grown in BHIS broth (Brigham & Malamy, 2005) at 37 °C in a Coy anaerobic chamber. *E. coli* Δ*rnf* was grown under different conditions: under aerobic conditions in LB (Miller) medium, LBNT LB-Miller medium (including 300 mM NaCl, 50 mM Tris-HCl, pH 8), LBK medium (LB-Miller in the presence of 50 mM KCl), minimal glucose medium (M9) and in the presence of 50 mM H_2_O_2_ and 1 mM paraquat, as well as under anaerobic conditions in minimal medium (M9) containing 0.04% yeast extract with either 27 mM glycerol or 50 mM Na-formate as electron donor and different electron acceptors (40 mM nitrate, 5 mM nitrite, 70 mM DMSO, 45 mM TMAO, or 50 mM fumarate). *V. cholerae* was grown in LB medium (pH 7) containing 170 mM NaCl.

### Preparation of washed membranes

Membranes were prepared as described (Imkamp et al., 2007) under strictly anaerobic conditions with some modifications. Cells were grown to the end of the exponential growth phase, harvested by centrifugation (11,300 × g at 4 °C; Contifuge Strato; Heraeus, Osterode, Germany) and washed twice with buffer A (50 mM Tris-HCl (pH 7.0), 20 mM MgSO_4_, 20% glycerol, 2 mM DTE (dithioerythritol) and 4 μM resazurin). The cells were resuspended in 15 ml buffer A and disrupted by a single passage through a French press (16,000 psi) or for *B. fragilis* by sonication under anaerobic conditions. Cell debris and whole cells were removed by one centrifugation step (23,700 × g, 20 min, 4 °C). The cell free extract was separated into cytoplasmic and membrane fraction by ultracentrifugation (190,000 × g, 1 h, 4 °C). The resulting sediment was washed in buffer A and membranes were again sedimented by an ultracentrifugation step (190,000 × g, 1 h, 4 °C). Membranes were resuspended in 5 ml buffer A and used immediately for the experiments. Protein concentrations were determined as described previously (Bradford, 1976).

### Preparation of inverted membrane vesicles of *C. ljungdahlii*

Cells were grown in a 20-l-scale as described above in MES-buffered complex medium containing 56 mM fructose. 20 g (wet weight) cells were resuspended in ∼150 ml buffer A (50 mM Tris, 20 mM MgSO_4_, 20% glycerol, 2 mM DTE, 4 μM resazurin, pH 7.5) and digested with 400 mg lysozyme at 37 °C for 20 minutes. Afterwards, the cells were passed once through a French Press at 41 MPa. A low speed centrifugation step (4500× g, 45 minutes, 4 °C) was succeeded by ultracentrifugation at 120,000 × g and 4 °C for another 45 minutes. The vesicles were washed once in buffer A and vesicles were sedimented again *via* ultracentrifugation as described above. The resulting pellet was resuspended in the same buffer in a volume of 5 ml.

### Measurement of Fd_red_:NAD^+^ oxidoreductase activity

Measurement of electron transfer from reduced ferredoxin to NAD^+^ by either membranes or inverted membrane vesicles was performed as described (Hess, Schuchmann & Müller, 2013) in anaerobic cuvettes filled with 1 ml 20 mM Tris-HCl buffer (pH 7.7) containing 2 mM DTE and 4 μM resazurin at a pressure of 0.5 × 10^5^ Pa CO. Ferredoxin (30 μM; purified from *C. pasteurianum* as described (Schönheit, Wäscher & Thauer, 1978)), Acs/CODH (30 μg/ml; purified from *A. woodii* as described (Hess, Schuchmann & Müller, 2013)), and washed membranes or inverted membrane vesicles (150 μg/ml) were added. If indicated, the ionophores ETH2120 or TCS were added at a concentration of 10 μM. The reaction was started by addition of NAD^+^ (4 mM). Formation of NADH was measured at 340 nm. Enzyme activity was measured at the same temperature at which the corresponding cells were grown. Na^+^ dependence of the electron transfer activity was measured as described (Hess, Schuchmann & Müller, 2013).

### Generation of the deletion mutant *B. fragilis* Δ*rnf*

We used the two-step procedure as previously described (Brigham & Malamy, 2005) for the isolation of chromosomal deletions in broad specificity hexokinase genes of *B. fragilis*. Briefly, a 480 bp PCR fragment beginning 300 bp upstream of *rnf*B and ending 180 bp into *rnf*B, was ligated to a 590 bp PCR fragment containing 260 bp of the 3′ end *rnf*A and 330 bp of adjacent sequence, then ligated into the cloning vector pYT102 to create plasmid pRAG394 in *E. coli*. A tri-parental mating was performed to transfer the pRAG394 construct into the chromosome of *B. fragilis* ADB77. The resulting co-integrates were verified by PCR analysis and candidates were subjected to the resolution protocol as described (Brigham & Malamy, 2005). Resolvants that contained the *rnf* deletion were identified by PCR analysis.

### Generation of the deletion mutant *E. coli* Δ*rnf*

The deletion of the *rnf* operon from the genome of *E. coli* was carried out using the Red-recombinase method as reported (Datsenko & Wanner, 2000). The parent strain was *E. coli* TOP-10 (Invitrogen). The *rnf* operon was replaced with a chloramphenicol cassette, which remained in the bacterial chromosome; thus the deletion strain is resistant to chloramphenicol up to 30 μg/ml. The deletion of the *rnf* operon was confirmed by PCR and by verifying the loss of fluorescent bands corresponding to RnfD and RnfG in SDS-PAGE membrane preparations.

## Results

**Figure 1:**
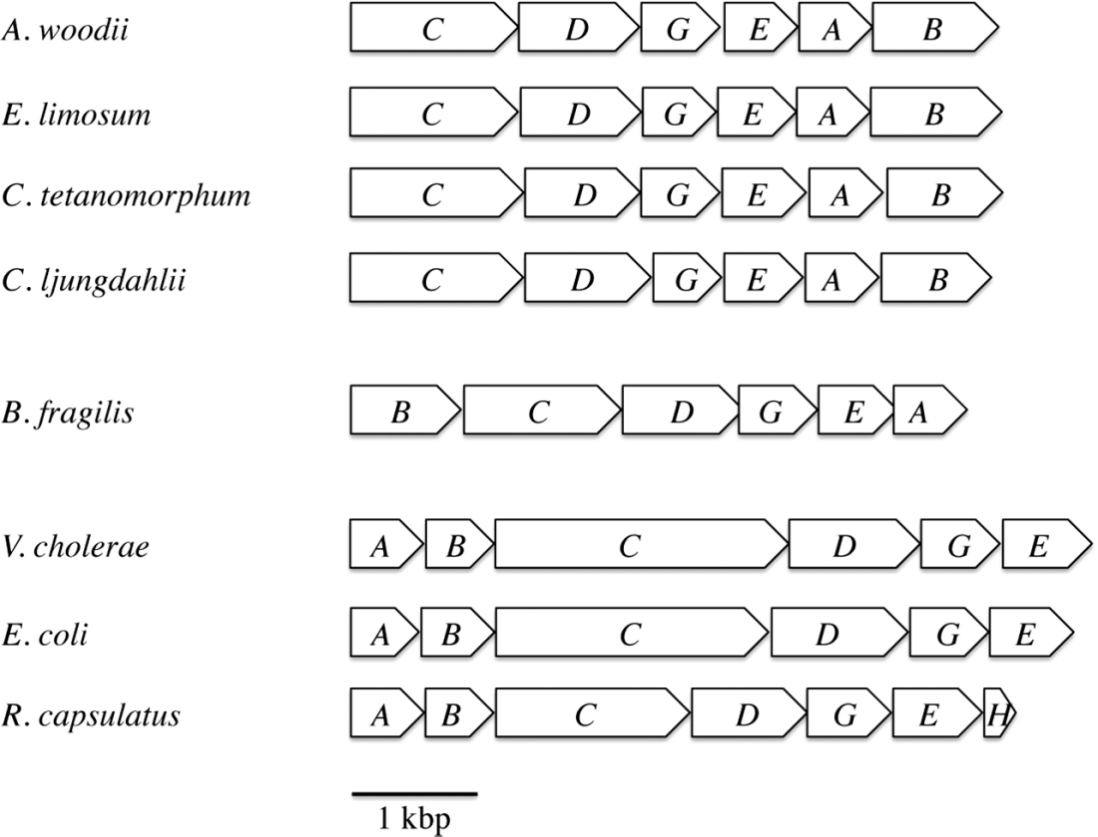
Arrangement of the *rnf* genes in different bacteria.

For the survey of ferredoxin:NAD^+^ oxidoreductase activity we used membranes of the strict anaerobes *A. woodii*, *C. tetanomorphum*, *C. ljungdahlii*, the nanoaerophile *B. fragilis*, and the facultative aerobes *R. capsulatus*, *V. cholerae* and *E. coli*. The genetic organization of the *rnf* genes in these species is shown in Fig. 1.

### *A. woodii*

As mentioned above, the acetogenic bacterium uses the Rnf complex as a membrane potential generator during growth on H_2_ + CO_2_ or fructose; and in the reverse reaction to drive the endergonic reduction of ferredoxin with electrons derived from NADH during heterotrophic growth on, for example, 2,3-butanediol, ethanol or lactate. As can be seen from Table 1, NAD^+^ reduction occurred at a rate of 50 ± 5 mU/mg protein and 53 ± 3 mU/mg protein, respectively, in membranes from cells grown on fructose or 2,3-butanediol, but the activity was highest in cells grown on ethanol 169 ± 8 mU/mg) or lactate (398 ± 21 mU/mg).

**Table 1:** Fd_red_:NAD^+^ oxidoreductase activities of washed membranes of different bacteria. To determine Na^+^ dependence, activity was measured in the absence and presence of 20 mM NaCl. The contaminating Na^+^ concentration in the absence of added NaCl was 68 to 110 μM.

Organism	Growth substrate	Fno activity without NaCl [mU/mg]	Fno activity with NaCl [mU/mg]
*A. woodii*	fructose	17 ± 3^a^	50 ± 5^a^
*A. woodii*	2,3-butanediol	16 ± 3^b^	53 ± 3^b^
*A. woodii*	ethanol	68 ± 15^b^	169 ± 8^b^
*A. woodii*	D,L-lactate	133 ± 19^b^	398 ± 21^b^
*E. limosum*	fructose	111 ± 14^c^	334 ± 26^c^
*C. tetanomorphum*	glutamate	421 ± 15^b^	900 ± 29^b^
*C. ljungdahlii*	fructose	306 ± 7^b^	256 ± 7^b^
*B. fragilis*	glucose + brain heart infusion	5.3 ± 0.7^b^	37 ± 5^b^
*B. fragilis* Δ*rnf*	glucose + brain heart infusion	4.2 ± 0.6^b^	4 ± 0.6^b^
*E. coli*	yeast extract	n.d.	0^b^
*R. capsulatus*	glucose + DMSO	n.d.	0^b^
*R. capsulatus*	malate (phototrophic)	n.d.	0^b^
*V. cholerae*	LB medium	8.2 ± 0.8^b^	7.1 ± 0.7^b^

**Notes:**

a Data from Hess, Schuchmann & Müller (2013).

b Results of the present study.

c Data from Jeong et al. (2015).

Fno, ferredoxin; NAD^+^, oxidoreductase activity; n.d., not determined. Each value is the mean from 3 replicates.

### *C. ljungdahlii*

**Figure 2:**
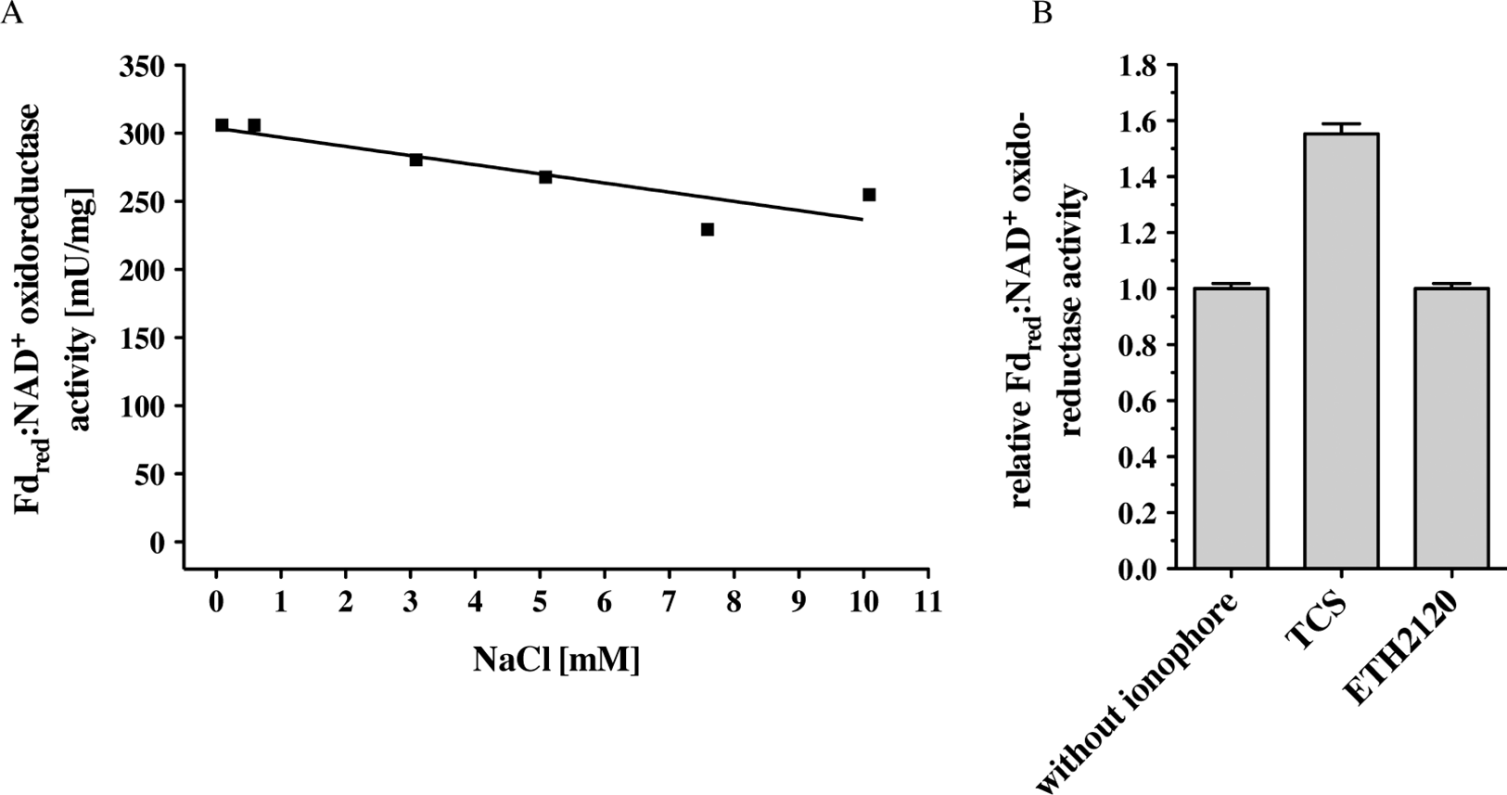
Fd_red_:NAD^+^ oxidoreductase activity of membranes of *C. ljungdahlii* as a function of Na^+^ concentration (A) and of inverted membrane vesicles in the presence of different ionophores (B). Fno activity was measured in anoxic cuvettes filled with 1 ml 20 mM Tris (sodium free)-HCl buffer (pH 7.7) containing 2 mM DTE and 2 μM resazurin at a pressure of 0.5 × 10^5^ Pa CO. NaCl was added to the concentration indicated. Ferredoxin (30 μM), Acs/CODH (30 μg/ml), and washed membranes or inverted membrane vesicles (150 μg/ml) were added. If indicated, the ionophores ETH2120 or TCS were added at a concentration of 10 μM. The reaction was started by addition of NAD^+^ (4 mM). Formation of NADH was measured at 340 nm.

*C. ljungdahlii* can grow autotrophically on H_2_ + CO_2_ or CO and produces acetate and ethanol. Inhibitor studies that had been done with whole cells were consistent with the presence of an enzyme that generates a primary electrochemical proton potential across the cytoplasmic membrane (Tremblay et al., 2012). Deletion of the *rnf* genes had resulted in a loss of the proton motive force which is consistent with the hypothesis of the presence of a proton-translocating Rnf complex in *C. ljungdahlii*. Indeed, membranes of *C. ljungdahlii* catalyzed electron transfer from reduced ferredoxin to NAD^+^ with an activity of 256 ± 7 mU/mg (Table 1). This activity was not dependent on Na^+^ (measured in a range of 68 μM to 10 mM NaCl) (Fig. 2A). Electron transfer from reduced ferredoxin to NAD^+^ in inverted membrane vesicles was stimulated by the protonophore TCS but not by the sodium ionophore ETH2120 (Fig. 2B). These data are consistent with the hypothesis that the Rnf complex of *C. ljungdahlii* uses H^+^ as coupling ion, not Na^+^. F_1_F_O_ (Rahlfs & Müller, 1997; Müller, 2003) and A_1_A_O_ (Müller, Rupert & Lemker, 1999) ATP synthases have a characteristic Na^+^ binding motif in their *c* subunits (Q/E in helix one, E/D and S/T in helix two). The absence of a Na^+^-binding motif in subunit *c* of the ATP synthase in *C. ljungdahlii* is in line with the hypothesis of a H^+^-Rnf.

### *C. tetanomorphum*

This obligate anaerobic, glutamate fermenting bacterium was quoted in review articles to have an Rnf complex that could be partially purified (Boiangiu et al., 2005; Buckel & Thauer, 2013). The ion specificity was not discussed but speculated to be Na^+^. However, the**se** data are not published. Membranes of *C. tetanomorphum* grown on glutamate catalyzed electron flow from reduced ferredoxin to NAD^+^ with a rate of 900 ± 29 mU/mg (Table 1), 18-fold higher than the rate observed in fructose-grown cells of *A. woodii*. Without additional Na^+^ the activity was only 431 ± 16 mU/mg, which is consistent with the hypothesis that the enzyme uses Na^+^ as coupling ion.

### *E. coli*

The *rnf* genes in *E. coli* are termed *rsx* (Koo et al., 2003) and have been shown to be upregulated during aerobic growth (Partridge et al., 2006). However, membranes from aerobically grown cells prepared under anaerobic conditions had no ferredoxin:NAD^+^-oxidoreductase activity. Furthermore, a deletion of the *rnf* operon from *E. coli* in the strain TOP10 showed no growth phenotype, neither under aerobic nor under anaerobic conditions.

### *R. capsulatus*

As mentioned above, the *rnf* genes were first described in *R. capsulatus* and hypothesized to be involved in nitrogen fixation by providing low potential electron for nitrogenase (Schmehl et al., 1993). Membranes of cells grown diazotrophically had no ferredoxin:NAD^+^-oxidoreductase, neither if the cells were grown in the light nor under respiratory conditions with glucose or DMSO.

### *B. fragilis*

The nanoaerophilic bacterium *B. fragilis* not only has *rnf* genes but also *nqr* and *nuo* (complex I) genes. Cytoplasmic membranes prepared from cells grown on complex medium catalyzed electron transfer from reduced ferredoxin to NAD^+^ with a rate of 37 ± 5 U/mg (Table 1). In the absence of NaCl, the activity was only 5.3 ± 0.7 mU/mg (Table 1), arguing for a Na^+^-dependence of the reaction. Since a genetic system is available for this organism, we deleted the *rnf* genes to establish the gene-polypetide correlation of the observed activity. Indeed, the ferredoxin:NAD^+^ oxidoreductase activity was reduced to 11% in the Δ*rnf* mutant in both the presence and absence of NaCl (Table 1), which is evidence that the majority of the ferredoxin:NAD^+^ oxidoreductase measured is encoded by the *rnf* genes.

### *V. cholerae*

The presence of *rnf* genes in *V. cholerae* was long known, however, a function of Rnf in this bacterium has not yet been described. Cytoplasmic membranes of *V. cholerae* catalyzed electron transfer from reduced ferredoxin to NAD^+^ with a rate of 7.1 ± 0.7 mU/mg (Table 1). As in *C. ljungdahlii*, this activity was independent of the Na^+^ concentration, arguing for a H^+^-dependent enzyme.

## Discussion

The assay described before to detect ferredoxin:NAD^+^ oxidoreductase activity in *A. woodii* (Hess, Schuchmann & Müller, 2013) was apparently suitable to detect the same activity in *C. ljungdahlii*, *C. tetanomorphum*, *B. fragilis*, and *V. cholerae*. This is actually all the more surprising since the assay requires that the ferredoxin:NAD^+^ oxidoreductase accepts electrons from *C. pasteurianum* ferredoxin. Not only must the redox potential be suitable but also the protein-protein interaction of the ferredoxin and its cognate receptor, most likely RnfB (Suharti et al., 2014) has to allow electron flow from the donor protein to the acceptor protein. The lack of activity in membranes of *E. coli* or *R. capsulatus* may be due to the differences in ferredoxin or simply reflect the fact that ferredoxin is not the electron carrier used by these enzyme complexes. Bacteria such as *A. woodii* encode several different ferredoxins but the one used by the ferredoxin:NAD^+^ oxidoreductase is not known. Therefore, phylogenetic analysis cannot be done. Thus far, the electron acceptor used by the Rnf complexes of *E. coli* and *R. capsulatus* remain to be identified. Moreover, the Δ*rnf* strain of *E. coli* has no phenotype and thus, the physiological role in *E. coli* also remains to be elucidated. Koo et al. (2003) has suggested that Rnf is the electron donor to the Fe-S protein SoxR that is part of a ROS sensor in the cell (Hidalgo, Ding & Demple, 1997). The role of Rnf would be to maintain SoxR in a reduced, and thus inactive state. In the presence of oxygen radicals, oxidized SoxR signals the cell to activate a response to ROS thus in the *rnf* deletion mutant, SoxR is expected to always be in the oxidized state and the cellular systems that protect the cell against ROS would always be activated. This would make it difficult to define phenotypic differences between the *rnf* deletion mutant and wild type *E. coli*. In our hands the growth curve for the deletion mutant was essentially the same as for wild type in both rich and minimal media, and inclusion of ROS producing substances such as paraquat and hydrogen peroxide did not reveal any clear differences. Preliminary expression data for *V. cholerae* suggest that Rnf is expressed at the same levels in several growth and infection conditions, suggesting that changes in the environment have little effect on the expression of *rnf*. However, Rnf seems to be essential for *V. cholerae* since we were not able to generate a Δ*rnf* deletion mutant.

Of interest is the finding that the *rnf* deletion in *B. fragilis* did not alter the anaerobic growth properties of this strain on BHIS medium (which contains glucose) when compared to the wild type parental strain. There are other annotated oxidoreductases in the *B. fragilis* genome coded for by the *nuo* and *nqr* operons whose functions may be redundant with *rnf*; this might also explain why 11% of the ferredoxin:NAD^+^ oxidoreductase activity could still be measured in the *rnf* deletion strain.

The data presented here show that the ferredoxin:NAD^+^ oxidoreductase activity in *A. woodii* is regulated, since growth on different substrates resulted in different activities. There was no correlation between activity and direction of electron flow in context of the metabolism. However, there was a correlation between activity and the number of cycles the complex has to pass for the phosphorylation of 1 ATP in the metabolism: When converting lactate, the Rnf complex has to catalyze 2.5 cycles for every ATP that is gained in this pathway (Weghoff, Bertsch & Müller, 2015). When converting ethanol, the phosphorylation of 1 ADP requires the Rnf complex to catalyze 1.3 cycles (Bertsch et al., 2015). The conversion of 2,3-butanediol only needs 0.85 cycles for every ATP (Hess et al., 2015) and degradation of fructose is predicted to require 0.11 Rnf cycles per ATP. The correlation of these values is in good agreement with the specific Rnf activities we observed for each of these substrates (Table 1). Thus, the more Rnf activity is necessary for the metabolism, the higher is the experimentally determined activity.

The highest ferredoxin:NAD^+^ oxidoreductase activity was found in *C. tetanomorphum*. Again, this would argue for a high electron transfer rate through Rnf during glutamate fermentation. The activity was stimulated by Na^+^ indicating that the Rnf complex uses Na^+^ as coupling ion. This is also consistent with the hypothesis of a Na^+^/glutamate symporter in *C. tetanomorphum* (Buckel & Thauer, 2013) as well as a V-type ATP synthase (encoded in the genome) with the *c* subunit having a Na^+^ binding motif in the second hairpin. In contrast, the activity was not Na^+^-dependent in the acetogen *C. ljungdahlii*. The observed stimulation of NAD^+^ reduction in inverted membrane vesicles by a protonophore clearly resembles the phenomenon of respiratory control, showing a strict coupling of electron transfer to the translocation of an ion across the membrane. The electrochemical potential established by the ion and charge translocation sets up a thermodynamic backup pressure and reduces the rate of electron flow (as well as the coupled ion flow). Dissipation of the ion gradient will release the thermodynamic backup pressure and stimulate electron flow. Since this was observed only with a protonophore but not a sodium ionophore it can be concluded that the coupling ion is a proton, not a sodium ion. As discussed before (Biegel et al., 2011), there is no reason to believe that the Rnf complex can only translocate Na^+^ as it does in *A. woodii*. Actually, other membrane transporters are known that can translocate either Na^+^ or H^+^, depending on the species. In the case of the F_1_F_O_ ATP synthase, only the exchange of two out of roughly 4,000 residues will change the ion specificity of the enzyme from H^+^ to Na^+^ (Meier et al., 2009).

The final proof that the Rnf complex is a redox-driven ion (Na^+^/H^+^ pump) still has to await purification of the complex and its reconstitution into liposomes. An equally important proof is presented here for the first time in bacteria: deletion of the *rnf* genes of *B. fragilis* reduced the ferredoxin:NAD^+^ oxidoreductase activity by 89%. Although the physiological role of the Rnf complex and the origin of the remaining 11% activity remains to be identified, this is clear evidence that the *rnf* genes encode the ferredoxin:NAD^+^ oxidoreductase activity and in line with the observation that a Δ*rnf* mutant of the archaeon *Methanosarcina acetivorans* is impaired in Na^+^ transport coupled to electron flow from reduced ferredoxin to heterodisulfide (Schlegel et al., 2012).

## Additional Information and Declarations

### Data Deposition

The following information was supplied regarding data availability: The research in this article did not generate any raw data.
